# Hu8F4-CAR T cells with mutated Fc spacer segment improve target-specificity and mediate anti-leukemia activity *in vivo*

**DOI:** 10.21203/rs.3.rs-3937972/v1

**Published:** 2024-02-19

**Authors:** Jeffrey Molldrem, Hong He, Rolando Vedia, Sijie Lu, Qiaochuan Li, Kathryn Cox, Lisa St. John, Anna Sergeeva, Karen Clise-Dwyer, Gheath Alatrash, Elizabeth Shpall, Qing Ma

**Affiliations:** The University of Texas MD Anderson Cancer Center; The University of Texas MD Anderson Cancer Center; The University of Texas MD Anderson Cancer Center; MD Anderson; The University of Texas MD Anderson Cancer Center; The University of Texas MD Anderson Cancer Center; The University of Texas MD Anderson Cancer Center; The University of Texas MD Anderson Cancer Center; University of Texas MD Anderson Cancer Center; The University of Texas MD Anderson Cancer Center; The University of Texas MD Anderson Cancer Center; The University of Texas MD Anderson Cancer Center

**Keywords:** PR1, Hu8F4, CAR-T cell, AML

## Abstract

Hu8F4 is a T cell receptor (TCR)-like antibody with high affinity for leukemia-associated antigen PR1/HLA-A2 epitope. Adapted into a chimeric antigen receptor (CAR) format, Hu8F4-CAR is comprised of the Hu8F4 scFv, the human IgG1 CH2CH3 extracellular spacer domain, a human CD28 costimulatory domain, and the human CD3ζ signaling domain. We have demonstrated high efficacy of Hu8F4-CAR-T cells against PR1/HLA-A2-expressing cell lines and leukemic blasts from AML patients *in vitro*. Previous studies have shown that modification of the Fc domains of IgG4 CH2CH3 spacer regions can eliminate activation-induced cell death and off-target killing mediated by mouse Fc gamma receptor (FcgR)-expressing cells. We generated Hu8F4-CAR(PQ) with mutated Fc receptor binding sites on the CH2 domain of Hu8F4-CAR to prevent unwanted interactions with FcgR-expressing cells *in vivo*. The primary human T cells transduced with Hu8F4-CAR(PQ) can specifically lyse HLA-A2^+^ PR1-expressing leukemia cell lines *in vitro*. Furthermore, both adult donor-derived and cord blood-derived Hu8F4-CAR(PQ)-T cells are active and can eliminate U937 leukemia cells in NSG mice. Herein, we demonstrate that modification of the IgG1-based spacer can eliminate Fc receptor-binding-induced adverse effects and Hu8F4-CAR(PQ)-T cells can kill leukemia *in vivo*.

## Introduction

The advent of chimeric antigen receptor (CAR) therapy has ushered in a wave of immunotherapeutic advancements, particularly in the treatment of hematologic malignancies(1–5). CAR therapy has achieved profound efficacy and multiple approvals by the US Food & Drug Administration, particularly in the form of therapies targeting CD19 and B-cell maturation antigen (BCMA) expressed in hematologic malignancies(6). In the case of acute myeloid leukemia (AML), our group has identified and characterized the leukemia-associated antigen, PR1, a nonameric, HLA-A2-restricted peptide derived from serine proteases proteinase 3 and neutrophil elastase (7–9). PR1 is overexpressed on myeloid leukemia cells, and we have demonstrated the ability and efficacy of targeting the PR1 peptide bound to HLA-A2 on AML blasts by using PR1 peptide as a vaccine in a clinical trial, PR1-specific cytotoxic T lymphocytes, using a first-in-class T cell receptor-like monoclonal antibody named Hu8F4, and a bispecific CD3/Hu8F4 antibody(10–14). Furthermore, we have previously reported on the development and *in vitro* efficacy of the Hu8F4 monoclonal antibody as a 2nd generation CAR therapy named Hu8F4-CAR(15).

In the last 30 years, the development of CAR therapies has encompassed a variety of different structural and signaling components each of which confer different capabilities and advantages(16–18). Hu8F4-CAR consists of the Hu8F4 scFv (single chain variable fragment) VL2 and VH regions, the human IgG1 CH2CH3 extracellular spacer domain, a transmembrane and intracellular human CD28 costimulatory signaling domain, and human CD3z intracellular signaling domain. We have demonstrated the efficacy of Hu8F4-CAR-T cells *in vitro* against PR1/HLA-A2-expressing cell line targets and leukemic blasts from AML patients. However, in a leukemia xenograft mouse model, Hu8F4-CAR-T cells lacked activity and the ability to persist *in vivo* (data not shown). Interestingly, others have reported that non-signaling extracellular spacer domains can trigger activation-induced cell death (AICD) upon binding of Fc gamma receptor (FcgR)-expressing cells *in vivo* leading to activation and death of the CAR-T cells, thus limiting their persistence and activity *in vivo*(19, 20). Additionally, such interactions with cells expressing FcgR *in vivo* could result in off-target killing by activated CAR-T cells. Previous studies have shown that modifying these Fc domains such as those within the IgG4 CH2CH3 regions to eliminate FcgR binding restored CAR *in vivo* antitumor effects and persistence(20). Since Hu8F4-CAR indeed contains an extracellular nonsignaling Fc domain of IgG1 (IgG1 CH2CH3), we mutated the CH2 domain within the spacer to prevent these interactions with FcgR-expressing cells *in vivo* and termed this mutated Hu8F4-CAR as Hu8F4-CAR(PQ). While others have reported the ability to abolish Fc domains in IgG4 to prevent activation-induced cell death, few have reported the ability to accomplish this in an IgG1-based CAR therapy. Herein, we report that abrogating FcgR-binding capabilities within the IgG1 spacer region preserves Hu8F4-CAR(PQ)’s specific recognition of PR1/HLA-A2 and is active against PR1-expressing targets *in vitro* and *in vivo*.

## Materials and Methods

### Generation of mutated Fc receptor binding sites on Human IgG1 CH2CH3 domain

Site-directed mutagenesis was conducted using two sets of primers to alter amino acid residues ELLG (IgG1 CH2CH3 amino acid (AA)# 6–9) to PVA and N (CH2CH3 AA# 180) to Q. ELLG mutagenesis primers were CCGTGCCCAGCACCTCCCGTGGCCGGAACCGTCAGTCTTC (sense) and GAAGACTGACGGTTCCGGCCACGGGAGGTGCTGGGCACGG (antisense). Q mutagenesis primers were GAGGAGCAGTACCAGAGCACGTACCGT (sense) and ACGGTACGTGCTCTGGTACTGCTCCTC (antisense). Mutagenesis was conducted using the QuikChange Lightning Site-Directed Mutagenesis Kit from Agilent Technologies (Santa Clara, CA). Mutagenesis was confirmed via Sanger sequencing.

### Generation of mutated Hu8F4-CAR(PQ)-T cells

Fc-mutated Hu8F4-CAR(PQ)-T cells were generated using healthy adult donor peripheral blood mononuclear cells (PBMCs) or umbilical cord blood mononuclear cells (CBMCs) obtained from the University of Texas MD Anderson Cancer Center Blood Bank. The mononuclear cells were isolated via density gradient centrifugation using Histopaque-1077 Hybri-Max from Sigma-Aldrich (St Louis, MO). Because HLA-A2^−^ (negative) cells are required to generate Hu8F4-CAR-T cells, HLA-A2 status of each donor was confirmed using 5μL FITC-conjugated anti-human HLA-A2 (clone BB7.2) antibody from BioLegend (San Diego, CA) and assessed on a flow cytometer. To activate the T cells, two days prior to transduction (Day − 2), 24-well non-tissue culture-treated plates were coated with 0.5mL per well of a 1μg/mL cocktail of anti-human CD3 (clone HIT3a) and anti-human CD28 (clone CD28.2) from BioLegend (San Diego, CA) each for four hours. Following this, each well was blocked with complete medium consisting of 223mL Click’s Medium from Irvine Scientific (Santa Ana, CA), 222mL of RPMI 1640 from Corning (Corning, NY), 50mL of heat-inactivated fetal bovine serum from Gibco (Waltham, MA), and 5mL penicillin/streptomycin solution from Cytiva (Marlborough, MA) for 30 minutes and 1 million HLA-A2^−^ (negative) PBMCs were plated per well and incubated at 37°C overnight. The following day (Day − 1), PBMCs were stimulated with 50IU/mL rhIL-2 from R&D Systems (Minneapolis, MN) and a separate 24-well non-tissue culture-treated plate was coated with 7μL human RetroNectin from Takara (Kusatsa, Japan) in 1mL of sterile PBS per well and incubated overnight at 4°C. The day of transduction (Day 0), RetroNectin solution was removed, blocked with 1mL complete medium for 30 minutes, and 2mL of Hu8F4-CAR(PQ)-packaged retrovirus supernatant was added to each well. Upon adding retrovirus to each well, the plate was centrifuged at 2000g for 90 minutes. Retroviral supernatant was aspirated and 2mL of complete CAR-T culture medium supplemented with 100IU/mL rhIL-2, and 0.25–0.75×10^6^/2mL/well activated and stimulated T cells were added to each RetroNectin- and retrovirus-coated well and centrifuged at 1000g for 30 minutes before beginning CAR-T culture. Medium was changed every 2–3 days with complete CAR-T culture medium supplemented with 100IU/mL rhIL-2. Transduction efficiency, phenotypic assessment, and cytotoxicity assay was conducted at day 7 post-transduction.

### Phenotypic assessment of Hu8F4-CAR(PQ)-T cells

Hu8F4-CAR(PQ)-T cell phenotypic assessment was measured using a panel of specific reagents including viability dye (Ghost UV450) from Cytek (San Diego, CA); BUV737-conjugated anti-human CD3 (clone UCHT1) from BD Horizon (Franklin, NJ); PerCP-Cy 5.5-conjugated CD4 (clone OKT4), Brilliant Violet 605-conjugated CD8 (clone SK1), PE-Cy 7-conjugated CD45RA (clone HI100), and APC-Cy 7-conjugated CCR7 (clone G043H7) all from BioLegend (San Diego, CA); Alexa Fluor 647-conjugated goat anti-human IgG F(ab’)_2_ from Jackson ImmunoResearch (West Grove, PA); PE-conjugated PR1/HLA-A2 tetramer (Baylor College of Medicine Tetramer Core Facility); and PE-conjugated CMV pp65/HLA-A2 tetramer. All samples were run on a BD LSR Fortessa cytometer (Franklin, NJ), analyzed using BD FlowJo software, and statistically analyzed using GraphPad Prism software.

### In vitro cytotoxic assessment of Hu8F4-CAR(PQ)-T cells

Freshly transduced Hu8F4-CAR(PQ)-T cells were assessed for functional cytotoxic activity via a calcein-AM-based cytotoxicity assay. Target cells included the U937 wildtype (PR1/HLA-A2^−^), U937 A2^+^ (HLA-A2-transduced, PR1^+^), and T2 cell line (TAP-deficient cell line able to present exogenous peptides). T2 target cells were pulsed with 30μg of either PR1 (VLQELNVTV) or HIVgag (SLYNTVATL) peptide, then labeled with 5μg/mL calcein-AM from Corning (Corning, NJ). A suspension of 50μL containing 5000 target cells in RPMI 1640 medium supplemented with 10% heat-inactivated fetal bovine serum and 1% penicillin/streptomycin were plated per well in a 96-well V-bottom plate. With the same suspension medium, 50μL of effector cells were co-incubated for 3.5 hours with target cells. Effector cell numbers depended on effector-to-target (E:T) cell ratios of either 8:1, 5:1, 4:1, 2:1, 1:1, 0.5:1, or 0.25:1. Cytotoxicity was measured via calcein-AM released from lysed cells into the supernatant via fluorescence reading (Ex 485nm / Em 528nm) on an Agilent BioTek Cytation3 plate reader (Winooski, VT). Control wells to calculate fluorescence in test wells included control wells bearing only calcein-AM labeled target cells (to measure spontaneous release of calcein-AM) and wells bearing only calcein-AM-labeled target cells with a Triton X-100 detergent from Sigma-Aldrich (St Louis, MO) added (to measure maximal release of calcein-AM). Cell lysis was calculated via the following formula: % lysis = ((test sample RFU – SR RFU)/(MR - SR)) * 100, where RFU = relative fluorescence unit, SR = spontaneous release, and MR = maximal release. Data was acquired on the BioTek Gen 5 software, analyzed via Microsoft Excel, and plotted using GraphPad Prism software.

### Leukemia xenograft model

NOD.Cg-Prkdcscid Il2rgtm1Wjl/SzJ (NSG) female mice from the Jackson Lab (Bar Harbor, ME) were housed at the University of Texas MD Anderson Cancer Center with an Institutional Animal Care and Use Committee (IACUC) approved protocol. U937 cells from ATCC (Manassas, VA) were transfected with the HLA-A2*0201 gene as described previously (21). Red-Fluc-GFP lentiviral particles were purchased from PerkinElmer (Waltham, MA) and U937-HLA-A2*0201 cells were transfected with the RediFect Red-Fluc-GFP lentiviral particles. Sort purified GFP^+^ cells (U937-A2-Luc/GFP) were maintained in complete medium RPMI 1640 supplemented with 10% v/v FBS from Gibco (Waltham, MA) and 1% v/v penicillin/streptomycin solution from Cytiva (Marlborough, MA) at 37°C in 5% CO2. 5 × 10^3^ U937-A2-Luc/GFP were intravenously (IV) injected into mice, and Hu8F4-CAR(PQ)-T cells or non-transduced T cells (control) were IV injected into mice 3 days later.

In vivo bioluminescence (BLI) was performed using a cooled charge-coupled device camera system (IVIS Imaging System 100 from Xenogen /Caliper Life Sciences, Alameda, CA) weekly after the inoculation of U937-A2-Luc/GFP cells. Mice were injected with 75 mg/kg D-luciferin (Beetle Luciferin Potassium Salt, Regis Technologies, Morton Grove, IL) in 100 μL PBS subcutaneously near the scapula and were placed in the light-tight chamber of the imaging system under isoflurane anesthesia. Dorsal luminescent images were acquired 10 minutes after D-luciferin injection. The total signal in the ROI (photons/s) was quantified using Living Image software (version 4.7; PerkinElmer. Inc). The same ROI was applied to all images acquired sequentially in a single-imaging session for a given mouse. The growth ratio was plotted against days after cell inoculation.

## Results

### Expression of Fc-mutated Hu8F4-CAR(PQ) on primary T cells from adult PBMCs

**Figure 1:**
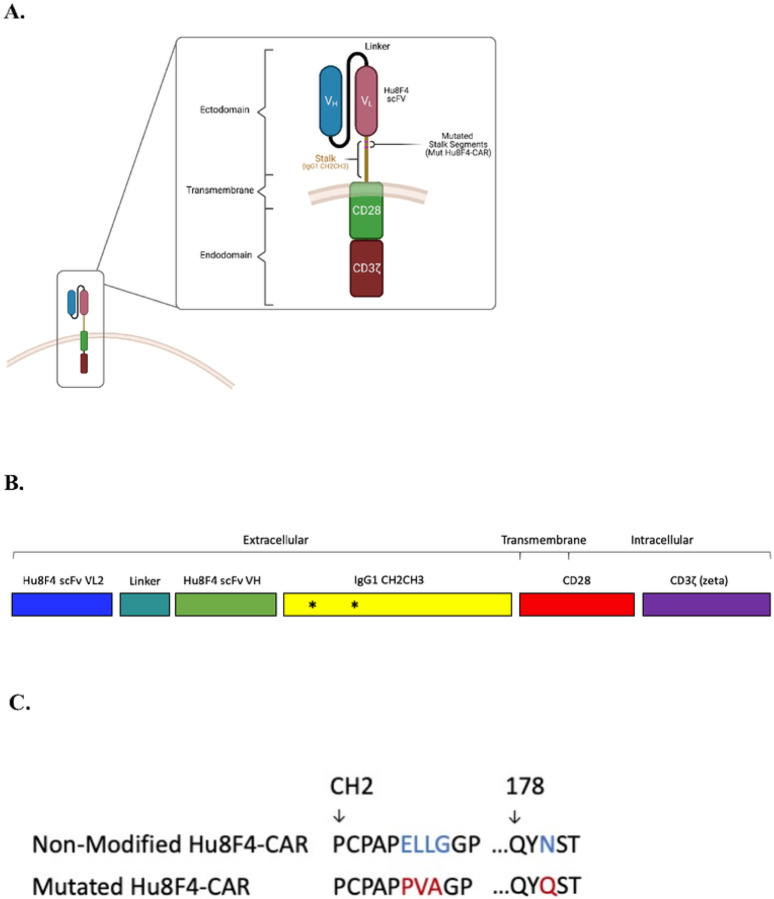
Hu8F4-CAR (PQ) -T construct. (A) Schematic of Hu8F4-CAR on the cell surface showing relative location of mutated Fc segments within CAR spacer/stalk. The mutant Hu8F4-CAR(PQ) construct consists of VL2 (variable region of Hu8F4 antibody light chain 2), VH (variable region of Hu8F4 antibody heavy chain), linker (GlyGlyGlyGlySer linker peptide), CH2CH3 (constant heavy region 2 and 3 of human IgG1), CD28 (human CD28 costimulatory domain), and zeta (human CD3 zeta signaling domain). (B) Linearized cassette view of Hu8F4-CAR regions. Asterisks mark relative location of Fc receptor mutations to abrogate Fc-mediated activation-induced cell death and allow *in vivo* activity and persistence. (C) Amino acid sequences demonstrating mutations introduced to abolish Fc domains. Figure 1A was created using BioRender.com.

We have previously described the development of a 2nd generation chimeric antigen receptor (CAR) against the leukemia-associated antigen, PR1/HLA-A2, named Hu8F4-CAR(15). Hu8F4-CAR consists of the Hu8F4 scFv VL2 and VH regions, the human IgG1 CH2CH3 extracellular spacer domain, a transmembrane and intracellular human CD28 costimulatory signaling domain, and human CD3z intracellular signaling domain (Fig. 1A). We have demonstrated the efficacy of Hu8F4-CAR *in vitro* on primary T cells against PR1/HLA-A2-expressing leukemia cell lines as well as blasts from AML patients. However, *in vivo*, Hu8F4-CAR T cells did not show *in vivo* activity in a leukemia xenograft model (data not shown). Others have reported the length of nonsignaling spacers/stalks exerted a major effect on CAR-T cell effector function after target engagement, and, more important, reported that spacers/stalks with Fc domains require alterations to prevent *in vivo* interactions with cells expressing Fc gamma receptors (FcgR) (20). They reported that CAR-T spacer Fc domain interaction with cells *in vivo* expressing Fc gamma receptors resulted in off-target activation of CAR-T cells and led to activation-induced cell death (AICD) of the CAR-T cells and limited their persistence and anti-tumor activity *in vivo* (20).

To generate mutated Fc receptor binding sites to prevent activation-induced cell death of CAR-T cells reported in IgG4 extracellular spacer domains(20), we conducted mutagenesis on Fc binding sites on the human IgG1 CH2CH3 extracellular spacer domain of Hu8F4-CAR (Fig. 1B). We replaced the four amino acids ELLG (Glu-Leu-Leu-Gly) of the IgG1 CH2 with tri-amino-acid PVA (Pro-Val-Ala) and mutated an additional N (Asn) to Q (Gln) (Fig. 1C), creating Hu8F4-CAR(PQ).

**Figure 2:**
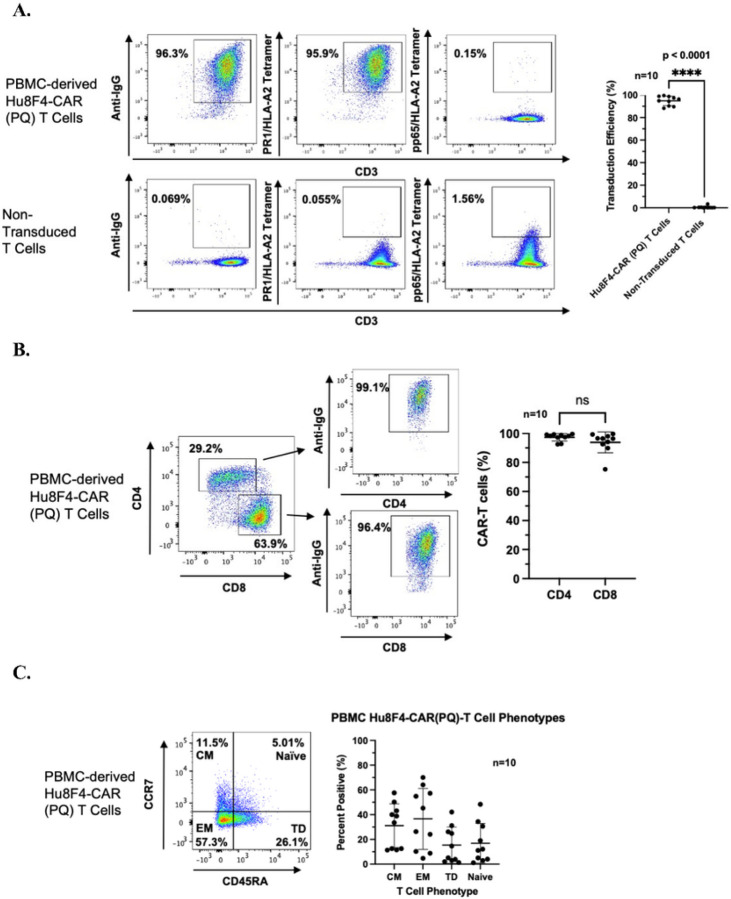
PBMC-derived T cells transduced with the Hu8F4-CAR (PQ) construct. (A) Hu8F4-CAR (PQ)-T cells are efficiently transduced compared to non-transduced T (NT) cells. Shown in the flow cytometry dot plots are representative figures using anti-human IgG which binds the CAR stalk and PR1/HLA-A2 tetramer, with pp65/HLA-A2 tetramer staining included as a tetramer control. Data points shown in graph on the right were assessed from cells stained positive for anti-human IgG antibody from 10 different experiments via unpaired, two-tailed Welch’s t test. (B) Hu8F4-CAR (PQ)-T cells are efficiently transduced in CD4 and CD8 populations with representative figures shown in flow cytometry dot plots. Data points shown in graph on the right were assessed from cells stained positive for anti-IgG antibody within CD4 and CD8 populations from 10 different experiments via unpaired, two-tailed Welch’s t test. (C) Hu8F4-CAR (PQ)-T cells primarily express memory T cell markers with representative figures shown in the dot plots. Data points shown in graph were assessed from cells stained positive for CD45RA and/or CCR7 from 10 different experiments. Error bars show standard deviation (SD).

To determine the function of Hu8F4-CAR(PQ), we transduced primary T cells from either healthy adult donor HLA-A2^−^ peripheral blood mononuclear cells (PBMC) or healthy umbilical cord blood mononuclear cells (CBMC) with retrovirus bearing Hu8F4-CAR(PQ) and measured transduction efficiency at day 7 post-transduction. We stained the cells with PR1/HLA-A2 tetramer, or CMV pp65/HLA-A2 tetramer as a control, and anti-human AffiniPure IgG F(ab’)_2_ which binds to the IgG1 spacer domain of Hu8F4-CAR. Data from 10 different experiments showed consistent, high transduction efficiency of Hu8F4-CAR(PQ) into primary T cells with a mean efficiency of 94.79% +/− 4.144% S.D (Fig. 2A; representative example shown in flow plots). Additionally, no differences were seen in the transduction efficiencies of CD4^+^ T cells compared to CD8^+^ T cells with both exhibiting mean transduction efficiencies greater than 93% (Fig. 2B). The majority of Hu8F4-CAR(PQ)-transduced adult T cells were of memory phenotype, with effector memory (EM) and central memory (CM) cells averaging 36.59% and 31.12% of the total transduced cells, respectively (Fig. 2C). This is in contrast to non-transduced cells which displayed higher frequencies of terminal differentiated (TD) effector T cells and naïve T cells (data not shown). The prominence of memory T cells within the Hu8F4-CAR(PQ) transduced population could further enhance activity, function, and long-term persistence of the cells *in vivo*.

**Figure 3:**
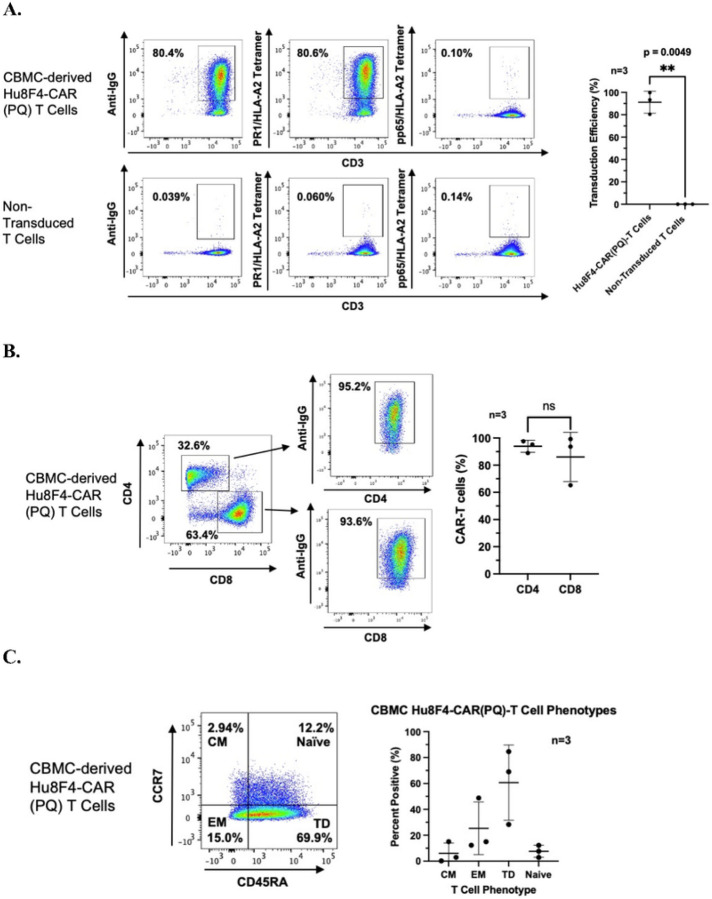
Human CBMC-derived T cells are efficiently transduced with the Hu8F4-CAR(PQ) construct. (A) CBMC-transduced Hu8F4-CAR (PQ)-T cells are efficiently transduced compared to non-transduced T cells. Shown in dot plot are representative figures using anti-human IgG which binds CAR stalk and PR1/HLA-A2 tetramer, with pp65/HLA-A2 tetramer staining included as a tetramer control. Data points shown in graph on the right were assessed from cells stained positive for anti-human IgG antibody from three different experiments via unpaired, two-tailed Welch’s ttest. (B) Shown in dot plot are representative figures. Data points shown in graph on the right were assessed from cells stained positive for anti-IgG antibody within CD4 and CD8 populations from three different experiments via unpaired, two-tailed Welch’s t test. (C) CBMC-transduced Hu8F4-CAR (PQ)-T cells primarily express effector T cell markers. Shown in dot plot is a representative figure. Data points shown in graph were assessed from cells stained positive for CD45RA and/or CCR7 from three different experiments. Error bars show standard deviation (SD).

In terms of Hu8F4-CAR(PQ)-T cells derived from umbilical CBMCs, our data from three independent experiments, each with a different healthy umbilical CBMC donor, also show consistent, high transduction efficiency of Hu8F4-CAR(PQ) into umbilical cord blood T cells (mean 91.20% +/− 9.885% S.D.) (Fig. 3A). As with the adult primary T cells, no significant differences were seen between the transduction efficiencies of CD4^+^ and CD8^+^ cord blood T cells (Fig. 3B). Phenotypically, umbilical CBMC-derived T cells transduced with Hu8F4-CAR(PQ) are primarily associated with effector phenotypes, predominantly TD effector and EM T cells (Fig. 3C). Not surprisingly, phenotypic differences were evident between Hu8F4-CAR(PQ)-T cells and non-transduced T cells (data not shown). Although there are differences, as noted above, on balance Hu8F4-CAR(PQ) is efficiently transduced and expressed in primary T cells and across phenotypically different T cell subsets.

### Hu8F4-CAR(PQ)-T cells actively and specifically target PR1-expressing cell lines in vitro

**Figure 4:**
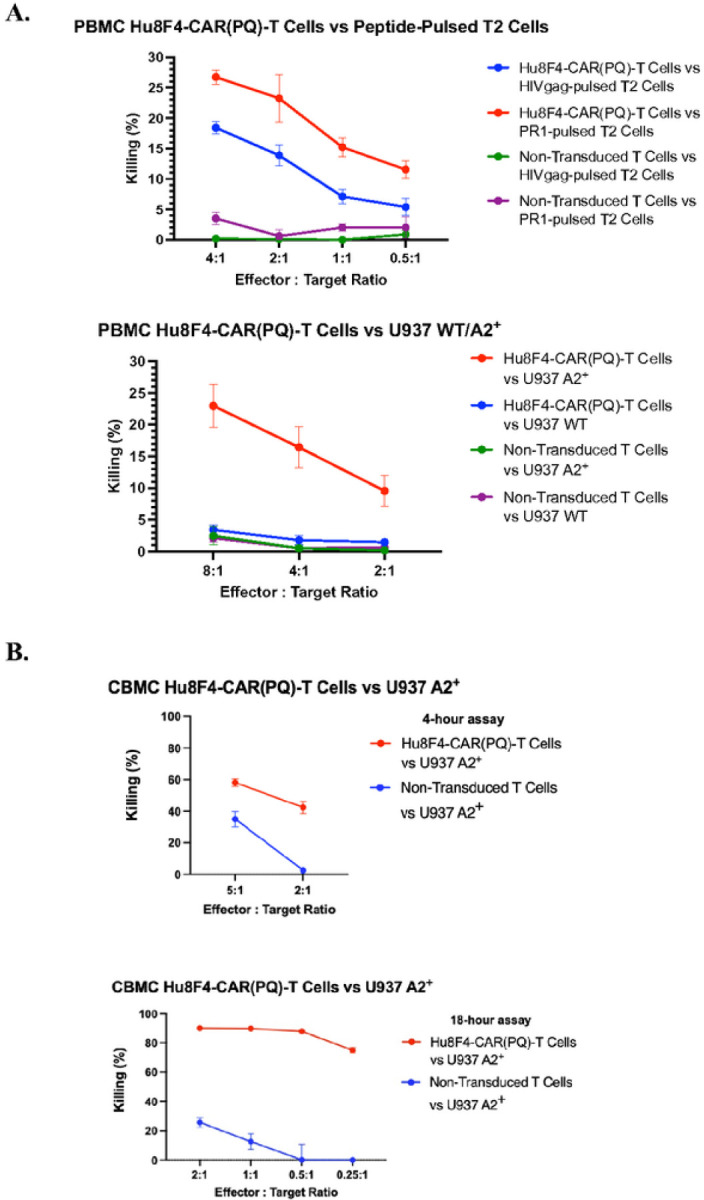
Adult PBMC- and umbilical CBMC-derived Hu8F4-CAR(PQ)-T cells demonstrate antigen-specific leukemia cell lysis in vitro. (A) PBMC-derived Hu8F4-CAR(PQ)-T cells show preferential killing of U937 A2^+^ cell line (top) which express PR1 and HLA-A2 compared to U937 WT cell line (bottom) which does not express PR1 or HLA-A2. Additionally, preferential killing is shown for T2 cell line pulsed with PR1 peptide compared to T2 cell line pulsed with HIVgag. U937 WT/A2^+^ cell line data is compiled from five different experiments, each with a different donor. Representative T2 cell line data is shown from three different experiments, each with a different donor. Each cytotoxicity assay was conducted after 3.5 hours. (B) CBMC-derived Hu8F4-CAR(PQ) cord blood T cells show preferential killing of U937 A2^+^ cell line compared to non-transduced T cells. Cytotoxicity data is from two different experiments, each with a different donor. Graph on the top shows cytotoxic activity after 4 hours and graph on the bottom shows activity after 18 hours.

To assess the functional activity of Hu8F4-CAR(PQ)-T cells after mutating the Fc binding capability in the CAR spacer domain, *in vitro* cytotoxicity assays were used to investigate whether Hu8F4-CAR(PQ)-T cells are active against PR1-expressing targets. To measure cytotoxic activity in PBMC-derived Hu8F4-CAR(PQ)-T cells, U937 WT (wildtype), U937-A2^+^ (HLA-A2-transduced), and the T2 cell line were used as targets. T2 cell line targets were pulsed with either PR1 peptide as specific target or HIVgag peptide as an irrelevant target control. Hu8F4-CAR(PQ)-T effector cells and targets were combined in effector-to-target ratios of either 8:1, 4:1, 2:1, 1:1, or 0.5:1 and co-incubated for 3.5 hours before measuring cell lysis. As shown in Fig. 4A, Hu8F4-CAR(PQ)-T cells show specific cytolytic activity against T2 cells pulsed with PR1 peptide as well as against the U937-A2^+^ cell line compared to T2 cells pulsed with HIVgag peptide and the U937 WT cell line, respectively. In addition, umbilical CBMC-derived Hu8F4-CAR(PQ)-T cells were assayed for cytotoxic activity against cell line targets. The U937 A2^+^ cell line was used as a target and CBMC-derived Hu8F4-CAR(PQ)-T cells or non-transduced T cells were used as effector cells in effector-to-target ratios of either 5:1 and 2:1or 2:1, 1:1, 0.5:1, and 0.25:1. As shown in Fig. 4B, there is strong activity of Hu8F4-CAR(PQ)-T cells against the U937-A2^+^ cell line. Altogether, Hu8F4-CAR(PQ) actively and specifically eliminates PR1-expressing cell line targets *in vitro*.

### PBMC-derived Hu8F4-CAR(PQ) -T cells eliminate U937 leukemia cells in NSG mice

**Figure 5:**
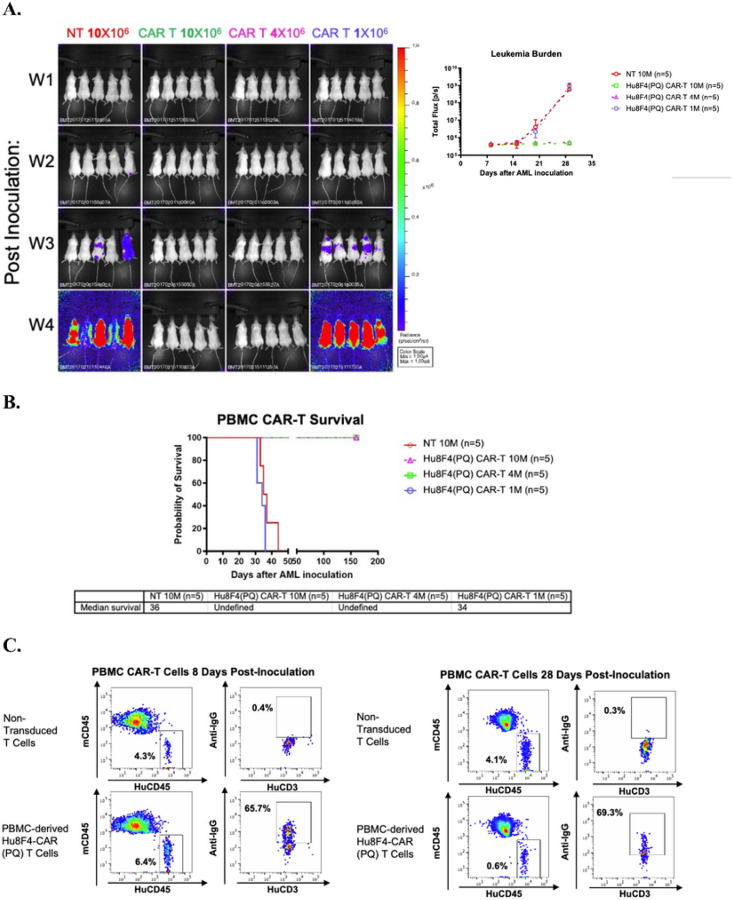
PBMC-derived Hu8F4-CAR(PQ)-T cells eliminate U937 leukemia cells and persist in NSG mice. NSG mice were sublethally irradiated and engrafted with U937-HLA-A2^+^ cells on day 0, and then treated with non-transduced control (NT) or various doses of Hu8F4-CAR(PQ)-T cells on day 3. (A) *In vivo* BLI of U937 leukemia cell-bearing mice treated with PBMC-derived Hu8F4-CAR(PQ)-T cells with all mice from a single experiment is displayed on week 1, 2, 3, and 4 post-infusion (left). Photons emitted from the U937-HLA-A2^+^-Fluc cells *in vivo* are shown over time for the indicated groups (right). (B) Survival curve and median survival data of U937-HLA-A2^+^-bearing NSG mice. (C) A representative data from the blood of Hu8F4-CAR(PQ)-T cell treated (10 million) (bottom panel) and NT mice (top panel) are shown on day 8 (left panel) and day 28 (right panel) post U937 cell infusion. Leukemia (human CD45^+^HLA-A2^+^) and CAR-T (human CD45^+^CD3^+^) cells were analyzed with FACS.

Using the established U937 leukemia/NSG mouse model in our lab, we evaluated whether the anti-tumor function of Hu8F4-CAR(PQ)-T cells is dose dependent *in vivo*. A bioluminescence (BLI) imaging system was used to systemically study spatial-temporal pattern of U937-A2^+^ cells transduced with a lentiviral vector encoding the firefly luciferase (Luc-GFP) genes (U937-A2^+^-Luc). U937-A2^+^-Luc cells (n = 5000 cells) were injected into NSG mice and monitored by BLI for 4 weeks post-infusion. Three different doses of PBMC-derived Hu8F4-CAR(PQ)-T cells were injected into the mice on day 3 when the U937-A2^+^-Luc leukemia cells had engrafted and were detectable with BLI. Mice that received 10 million non-transduced T cells (NT), as a control, showed persistent engraftment of leukemia while mice receiving Hu8F4-CAR(PQ)-T cells displayed a significantly reduced leukemia burden (Fig. 5A). The overall survival of U937-A2^+^-Luc-bearing mice was increased after treatment with higher cells doses of Hu8F4-CAR-T cells (4 and 10 million cells). Furthermore, PBMC-derived Hu8F4-CAR(PQ)-T cells can eliminate leukemia cells and prolong the overall survival of U937-A2^+^-Luc -bearing mice (Fig. 5B). The Hu8F4-CAR(PQ)-T (human CD45^+^CD3^+^) cells are present and detectable both 8- and 28-days post-infusion in blood (Fig. 5C). This data demonstrates that Hu8F4-CAR(PQ)-T cells exhibit specific antitumor effects and persist *in vivo*.

### Expanded CBMC-derived Hu8F4-CAR(PQ)-T cells are active against U937 leukemia cells in vivo.

**Figure 6:**
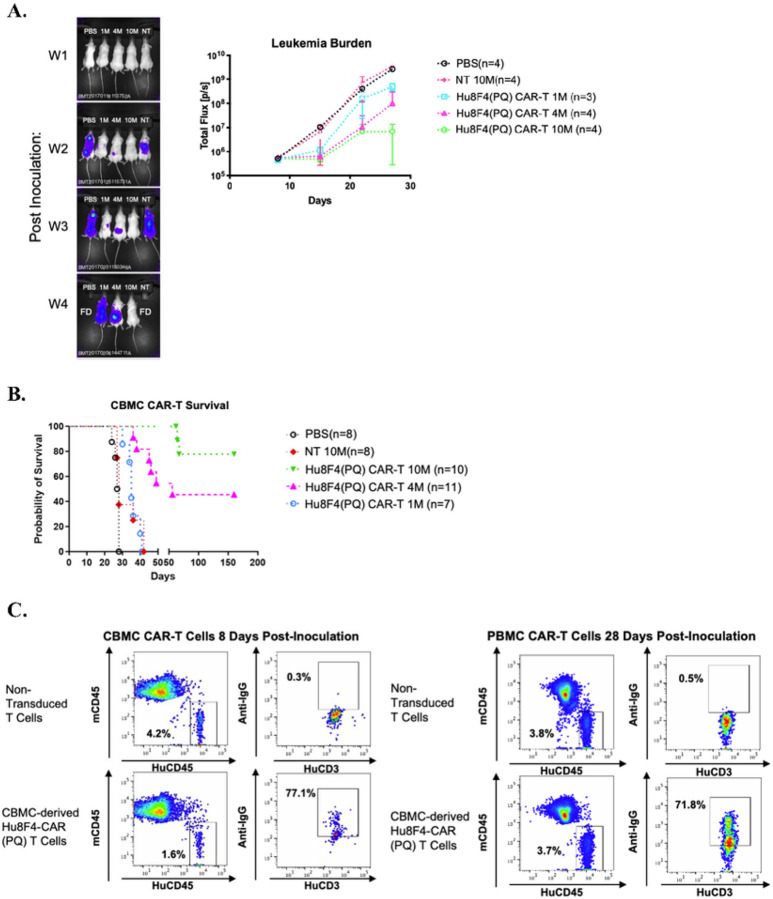
CBMC-derived Hu8F4-CAR(PQ)-T cells can reduce U937 leukemia burden in vivo. Hu8F4-CAR(PQ)-transduced T cells collected from two different CBMC-donors were infused into NSG mice engrafted with U937-HLA-A2^+^ cells, in addition to PBS and NT. (A) Quantification of dorsal view of representative *in vivo* BLI of U937 leukemia cell-bearing mice is displayed on week 1, 2, 3, and 4 post-infusion (left). Photons emitted from the U937- HLA-A2^+^-Fluc cells *in vivo* are shown over time for the indicated groups (right). (B) Survival curve and median survival data of U937-HLA-A2^+^-bearing NSG mice (lower right). (C) A representative data from the blood of Hu8F4-CAR(PQ)-T cell treated (10 million) (bottom panel) and NT mice (upper panel) are shown on day 8 (left panel) and day 28 (right panel) post U937 cell infusion. Leukemia (human CD45^+^HLA-A2^+^) and CAR- T (human CD45^+^CD3^+^) cells were analyzed with FACS.

Previously we have shown that human umbilical CBMC-derived Hu8F4-CAR T cells were capable of killing leukemia cell lines *in vitro* (15). In the current study, Hu8F4-CAR(PQ)-T cells were generated from three different CBMC-donors and were subsequently infused into NSG mice with established U937-A2^+^-Luc leukemia (Fig. 6). By week two, we can detect leukemia cell expansion in control mice (PBS and NT) by BLI (Fig. 6A). Similar results were seen with CBMC-derived Hu8F4-CAR(PQ)-transduced T cells from another donor (data not shown). Mice treated with 1 and 4 million of CBMC-derived Hu8F4-CAR(PQ)-T cells showed much slower expansion of U937-A2^+^-Luc cells. More importantly, mice treated with 10 million CBMC-derived Hu8F4-CAR-T cells did not have any detectable U937-A2^+^-Luc cells by week four and exhibited prolonged survival (Fig. 6B). We can detect the CBMC-derived Hu8F4-CAR(PQ)-T (human CD45^+^CD3^+^) cells both 8- and 28-days post-infusion in blood (Fig. 6C). In conclusion, we have demonstrated that CBMC-derived Hu8F4-CAR(PQ)-T cells can protect mice against U937-A2^+^-Luc leukemia cells, confer a significant survival advantage, and persist up to 28 days, a marked improvement from the initial Hu8F4-CAR construct, prior to Fc domain mutation.

## Discussion

We generated a Hu8F4-CAR(PQ) construct with mutated Fc receptor binding sites on the CH2 domain of Hu8F4-CAR and demonstrated consistent and high transduction efficiency of the new construct into both CD4^+^ and CD8^+^ T cells. Our *in vitro* cytotoxicity data shows potent and specific activity against PR1/HLA-A2^+^ targets. More importantly, Hu8F4-CAR(PQ)-T cells demonstrate strong anti-leukemic efficacy *in vivo*.

We modified the original Hu8F4-CAR design to resolve the lack of *in vivo* activity while maintaining the specificity and functionality of the CAR-T cells *in vivo*. One potential route to modify Hu8F4-CAR’s design was described in the findings Hudecek and others who reported the ability to restore *in vivo* function and persistence by mutating Fc-receptor binding domains with IgG-based spacer domains to prevent AICD and off-target activation(20, 22). Use of human IgG (IgG1 and IgG4)-derived hinge region plus heavy chain constant regions (CH2CH3) as the extracellular spacer domain is a common practice in CAR design. These modifications allow for straightforward detection of CAR expression using anti-Fc antibodies as well as flexible length adjustability by removing either CH2 and/or CH3 domains while still maintaining a low immunogenicity profile. Countering these advantages are the potential undesirable effects such as Fc-binding by FcgR-bearing cells, leading to off-target activation of CAR-T cells and non-specific target killing. In addition, antigen-independent activation due to binding of FcgR-expressing cells could result in AICD of the CAR-T cells and diminishing long term anti-tumor activity in vivo (20).

Hudecek et al. described a modification of an ROR1-specific CAR with an IgG4 constant heavy chains 2 and 3 (CH2CH3)-derived spacer (20). They replaced the known FcgR binding site in the CH2 of IgG4 with an IgG2 sequence that lacked such interaction. A similar effect was also observed in an earlier study with an IgG1-based anti-CD30-CAR by another group (22). Subsequently, Hudecek abrogated a conserved N-glycosylation site in the CH2 domain, which was implicated in additional FcgR binding by human monocytes, through single amino acid replacement (N into Q). The resulting modified ROR1-CAR maintained both target-specific cytolytic activity in vitro as well as persistent anti-tumor functionality in an *in vivo* xenograft model (20). We took advantage of the highly conserved amino acid sequences among the different classes of human IgG CH2 domains and modified the Hu8F4-CAR’s IgG1(CH2-CH3) spacer domain by replacing the known FcgR-binding site in IgG1 CH2 (ELLP) with the IgG2 CH2 sequence PVA, which reduced binding to FcgRI by 10^4^-fold (22). Following the strategy of others in IgG4 spacer domains, we also eliminated the N-glycosylation site, which is conserved between IgG1 and IgG4 (20).

The redesigned Hu8F4-CAR(PQ) construct maintains full cytotoxic activity *in vitro* against cell lines expressing PR1/HLA-A2 and is consistently and highly transduced into both CD4^+^ and CD8 + T cells. Interestingly, Hu8F4-CAR-transduced T cells derived from PBMCs show a preference towards memory phenotypes while non-transduced control T cells seem to show a diversity in T cell phenotypes, primarily naïve and terminally differentiated effector. Contrary to the original, unmodified Hu8F4-CAR T cells, which failed to sustain anti-leukemic activity in xenograft mice (data not shown), the Hu8F4-CAR(PQ)-T cells exhibited robust anti-leukemic activity *in vivo* at multiple dose levels. Notably high doses of Hu8F4-CAR(PQ)-T cells (10 million) could eliminate established leukemia (U937-A2^+^) completely. Decreased tumor burden correlated with the presence of Hu8F4-CAR(PQ)-transduced T cells in mouse peripheral blood taken during the early time points after the injection, suggesting that the modifications made in the IgG1 spacer region led to a more sustainable CAR-T cell population. *In vivo* anti-tumor function of the modified Hu8F4-CAR(PQ)-T cells was evident not only with adult PBMC but also with CBMC, providing alternative cell source options for use in clinical settings.

In this study, we have shown that CAR-T cells using human IgG1-based spacer can be modified to eliminate Fc receptor-binding-induced adverse effects on its survival and functionality *in vivo*. This paves the way for using such CAR-T cell products in future clinical trials against cancers. Furthermore, Hu8F4-CAR(PQ)-T cells demonstrate consistent high transduction efficiency into both PBMC- and CBMC-derived T cells and are active against PR1-expressing leukemic targets *in vitro* and *in vivo*.
